# Supplementary material for: Linked origins but distinct roles for extreme length and sequence variation at a tandem repeat in CACNA1C
Source: bioRxiv. 2026 Apr 23:2026.04.23.720446. Preprint. [Version 1] doi: 10.64898/2026.04.23.720446 (PMC13131567; doi:10.64898/2026.04.23.720446)
Supplement: Supplement 1 [file media-1.pdf]

Supplement: Linked origins but distinct roles for extreme length and sequence variation at a tandem repeat in *CACNA1C*

Janet H.T. Song[a,*], Vivien Zhao[a], Rachel L. Grant[b], David M. Kingsley [b,c,*]

[a] Department of Human Evolutionary Biology, Harvard University, Cambridge, MA, 02138, USA

[b] Department of Developmental Biology, Stanford University, Stanford, CA, 94305, USA

[c] Howard Hughes Medical Institute, Stanford University, Stanford, CA, 94305, USA

[*] Corresponding authors: janetsong@fas.harvard.edu, kingsley@stanford.edu

Supplemental Tables

Table S1: **30-bp variants**
Table S2: **Proportion of 30-bp variants in individuals in 1000 Genomes Project**
Table S3: **SNPs in linkage disequilibrium with TRACT^{L}**

Supplemental Figures

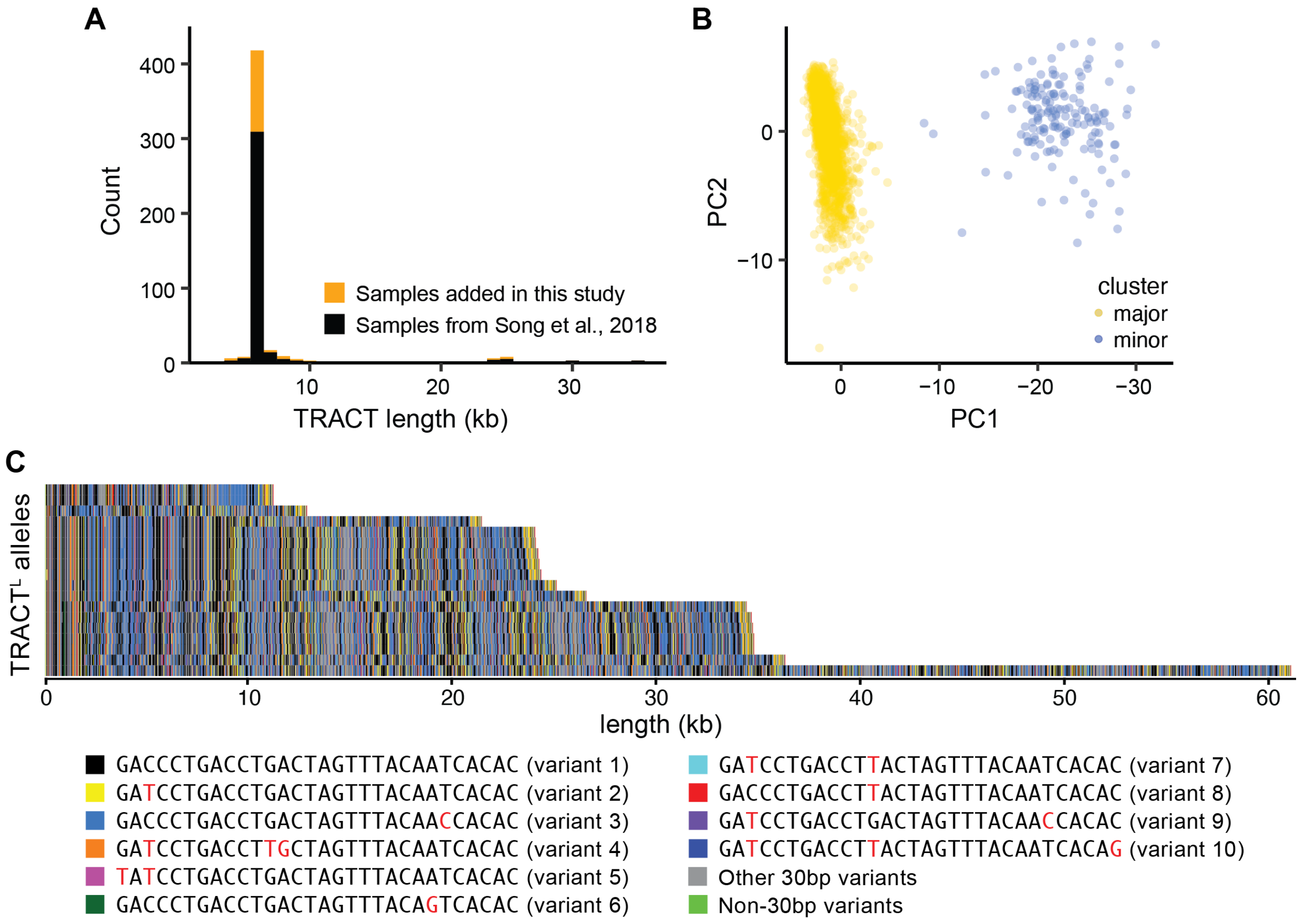

Figure S1: TRACT length and sequence variation. (A) Distribution of TRACT length by Southern blot for 362 alleles from (Song et al., 2018) in black and 130 alleles added in this study in orange. (B) PC1 (11.5% of the variance) and PC2 (2.8% of the variance) of 30-bp variant proportions for individuals in the 1000 Genomes Project (1000 Genomes Project Consortium, 2015). (C) Visualization of all $TRACT^L$ alleles from HGSVC (Logsdon et al., 2025), HPRC (Liao et al., 2023), and the Platinum Pedigree (Kronenberg et al., 2025) with coloring of the ten most common 30-bp variants.

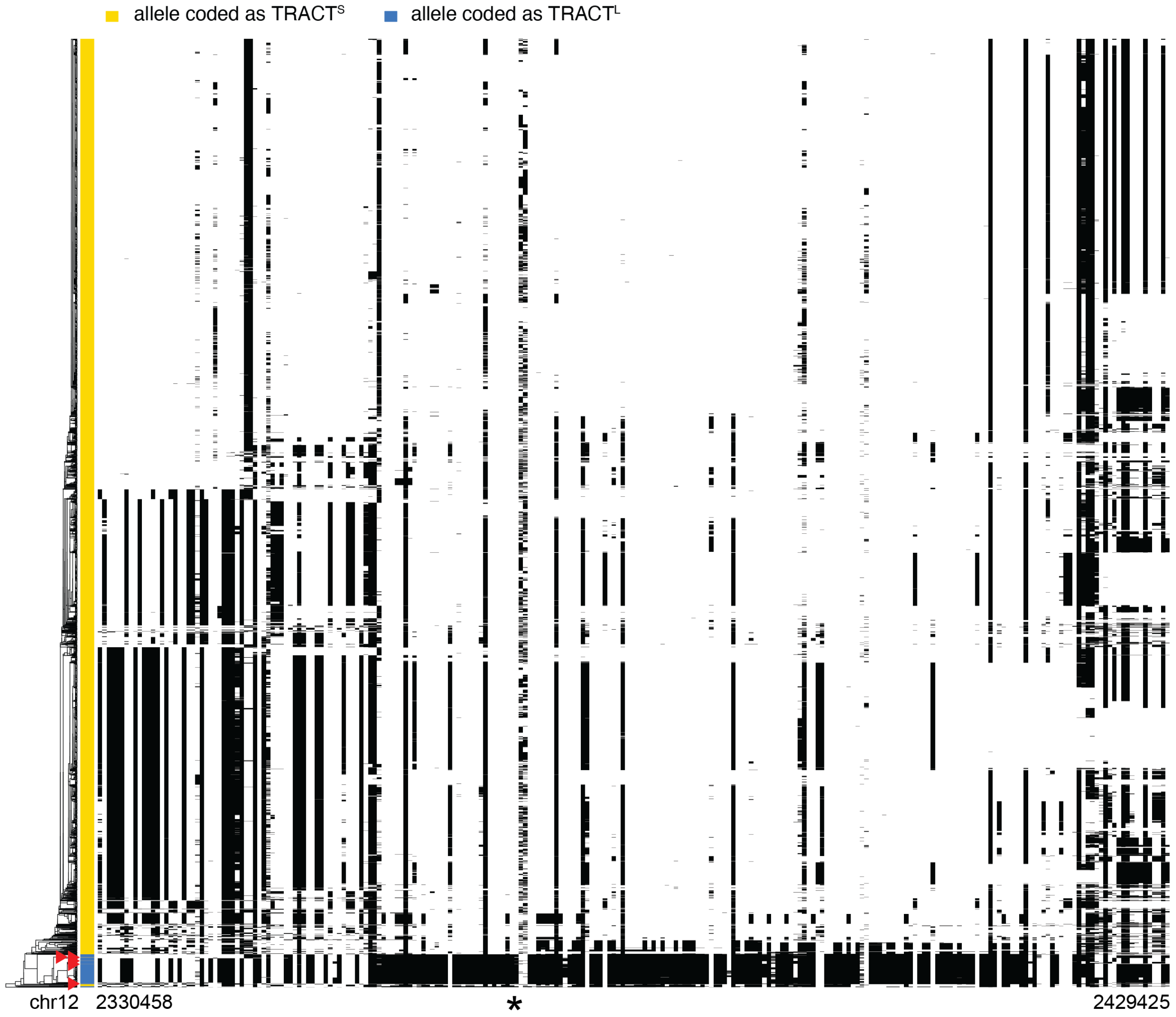

Figure S2: TRACT^{L} is found on one haplotype in the 1000 Genomes Project. TRACT^{S}/TRACT^{L} was coded as a variant for individuals in the 1000 Genomes Project, and the 2 Mb interval surrounding TRACT was phased using Beagle 5.0 (Browning and Browning, 2007) (Materials and Methods). The phased alleles were visualized using Haplostrips (Marnetto and Huerta-Sánchez, 2017) for chr12:2330458-2429425 (hg19). The genomic position of TRACT is indicated by an asterisk. Five red arrowheads indicate alleles from individuals that are likely homozygous for TRACT^{L}.

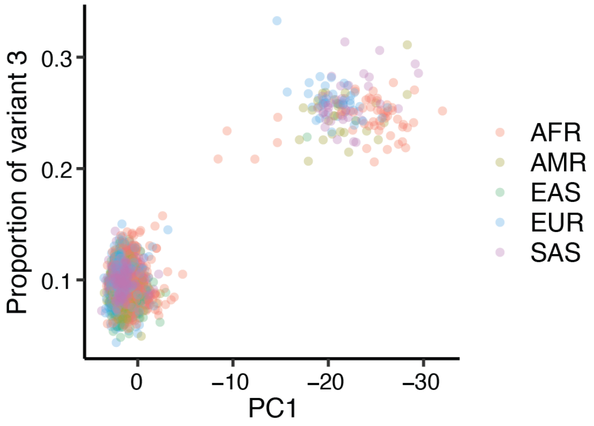

Figure S3: TRACT^{L} is found in every super population in the 1000 Genomes Project. Same plot as Fig. 1B colored by super population. AFR: Africans, AMR: Ad-Mixed Americans, EAS: East Asians, EUR: Europeans, SAS: South Asians.

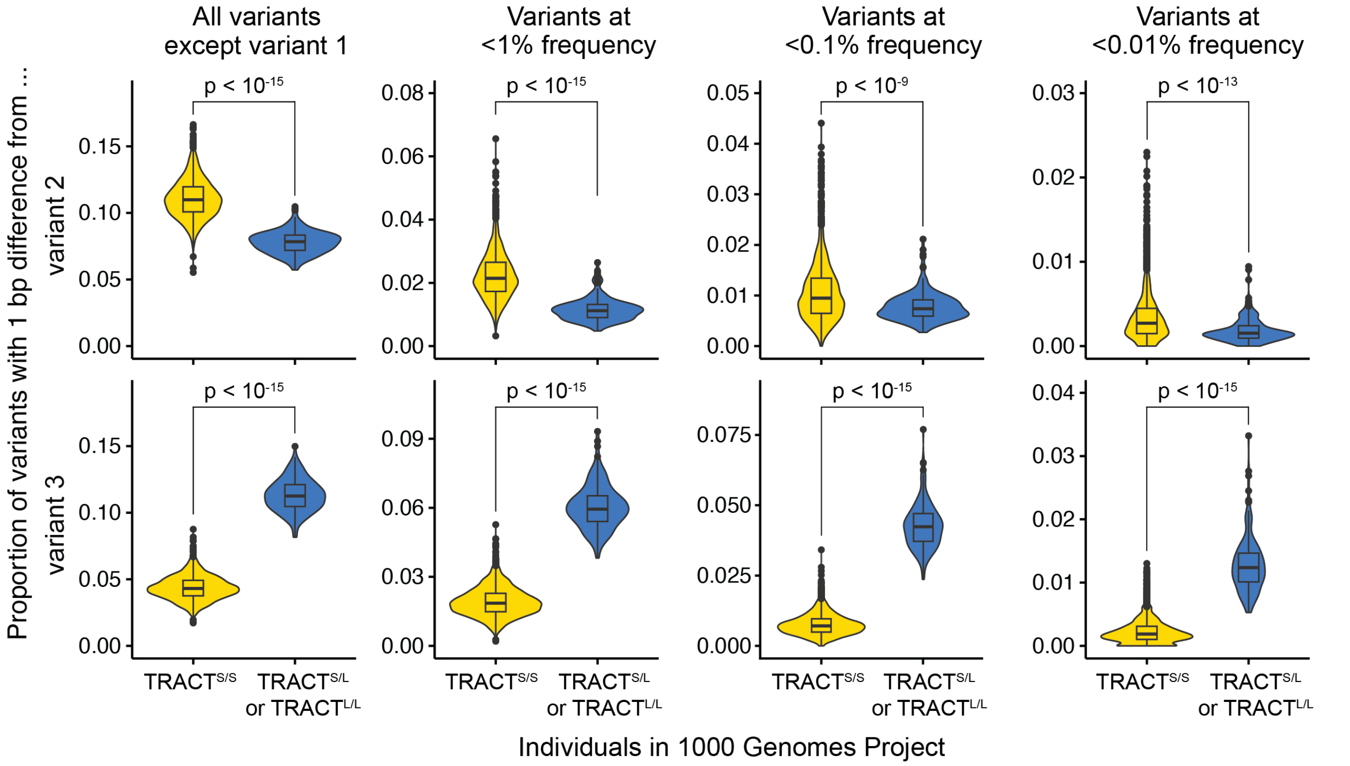

Figure S4: Proportion of 30-bp variants with 1 bp sequence difference from variant 2 or 3. Proportion of all variants except variant 1, variants with <1% frequency, variants with <0.1% frequency, and variants with <0.01% frequency that have a 1 bp sequence difference from variant 2 (top) or variant 3 (bottom) for TRACT$^{S/S}$ individuals (N = 2334) and TRACT$^{S/L}$ or TRACT$^{L/L}$ individuals (N = 156) in the 1000 Genomes Project. Statistical significance was assessed with the Wilcoxon rank-sum test.

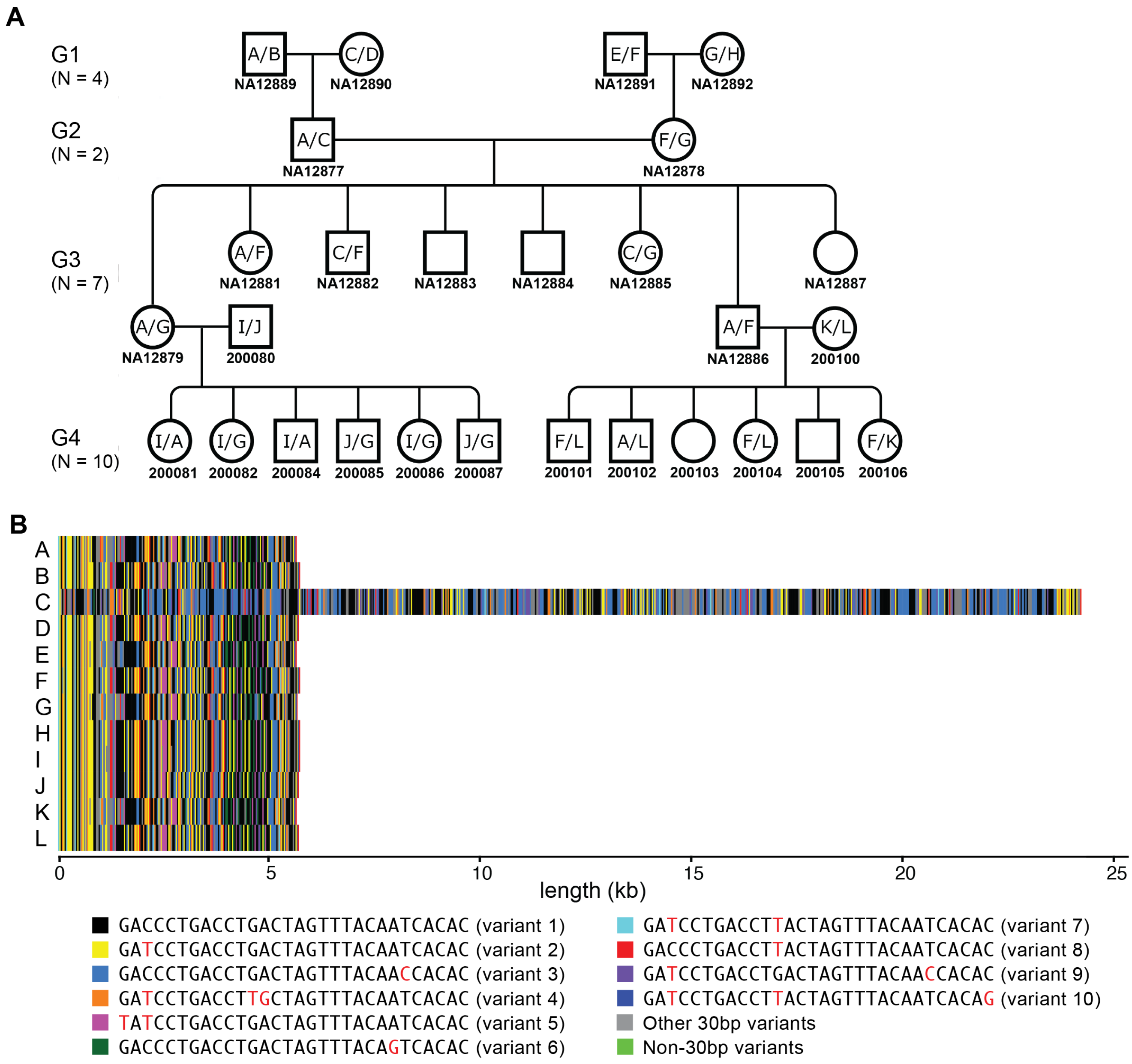

Figure S5: No evidence for TRACT germline instability in the Platinum Pedigree. (A) Alleles (labeled A-L) segregate as expected in a four-generation pedigree. No variation in TRACT length was detected. Data were not readily available for unlabeled individuals in the pedigree. (B) Visualization of all alleles found in the pedigree, with coloring of the ten most common variants.

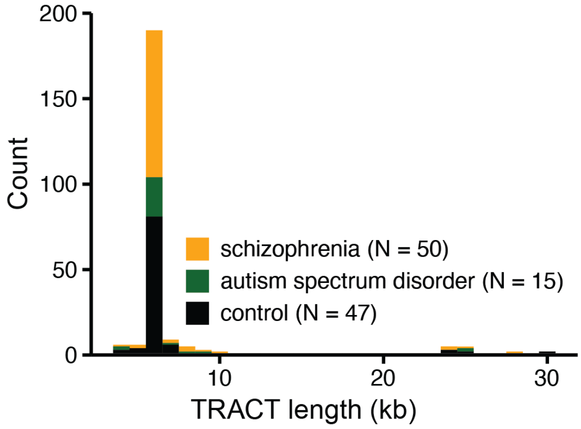

Figure S6: Distribution of TRACT length in individuals assayed for somatic mosaicism. Stacked distribution of TRACT allele lengths for 47 controls (black), 50 individuals with SCZ (orange), and 15 individuals with autism (dark green), as assessed by Southern blot.

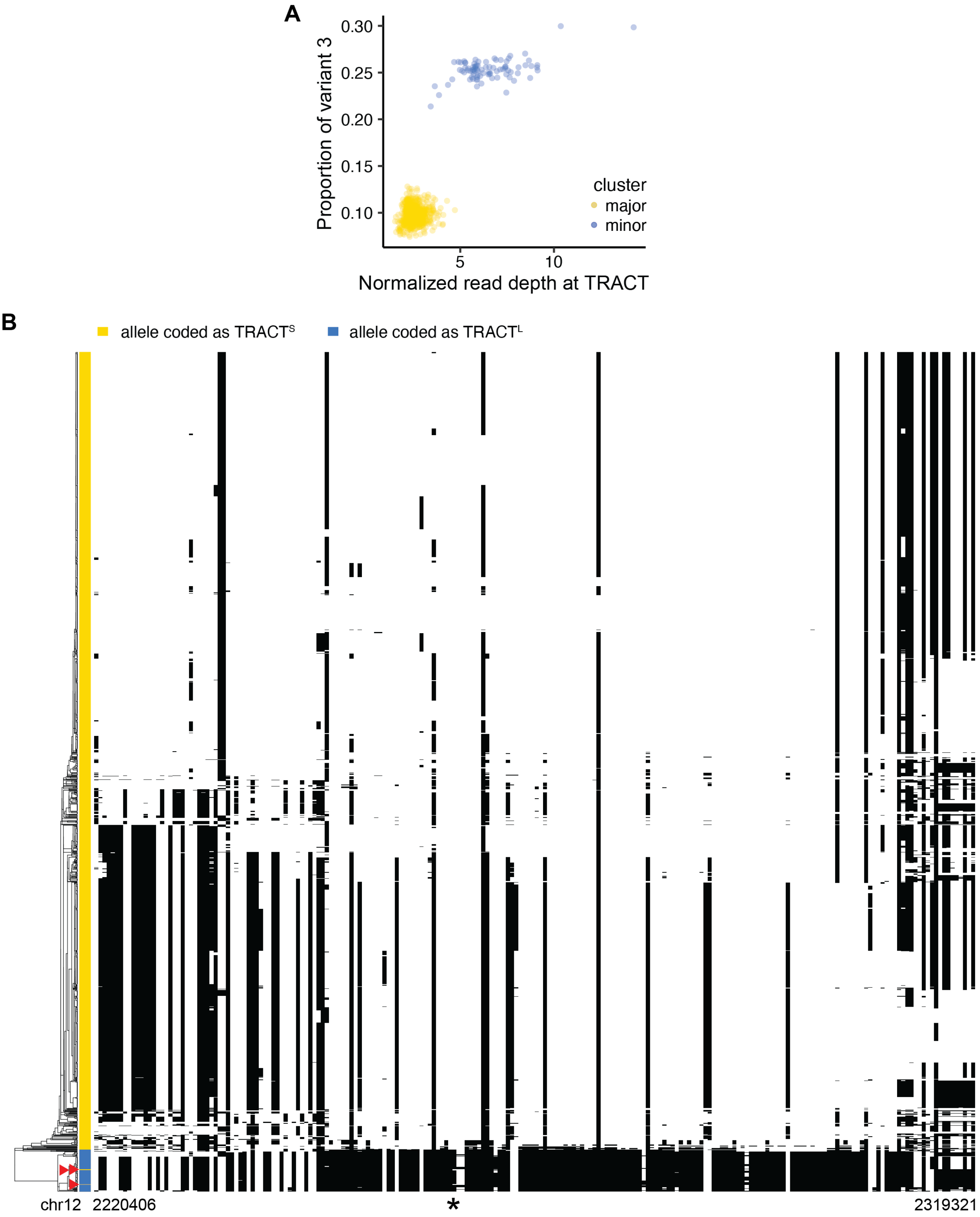

Figure S7: TRACT^L is found on one haplotype in GTEx. (A) Individuals in GTEx (Lonsdale et al., 2013) with high variant 3 proportion are enriched for increased read depth ($p < 10^{-65}$, 2-sample Kolmogorov–Smirnov test). The x-axis is the number of reads that map to TRACT divided by the total number of reads in each sample $x10^6$. (B) TRACT^S/TRACT^L was coded as a variant, and the 2 Mb interval surrounding TRACT was phased using Beagle 5.0 (Browning and Browning, 2007) (Materials and Methods). The phased alleles were visualized using Haplostrips (Marnetto and Huerta-Sánchez, 2017) for chr12: 2220406-2319321 (hg38). The genomic position of TRACT is indicated by an asterisk. Three red arrowheads indicate alleles from individuals that are likely homozygous for TRACT^L.

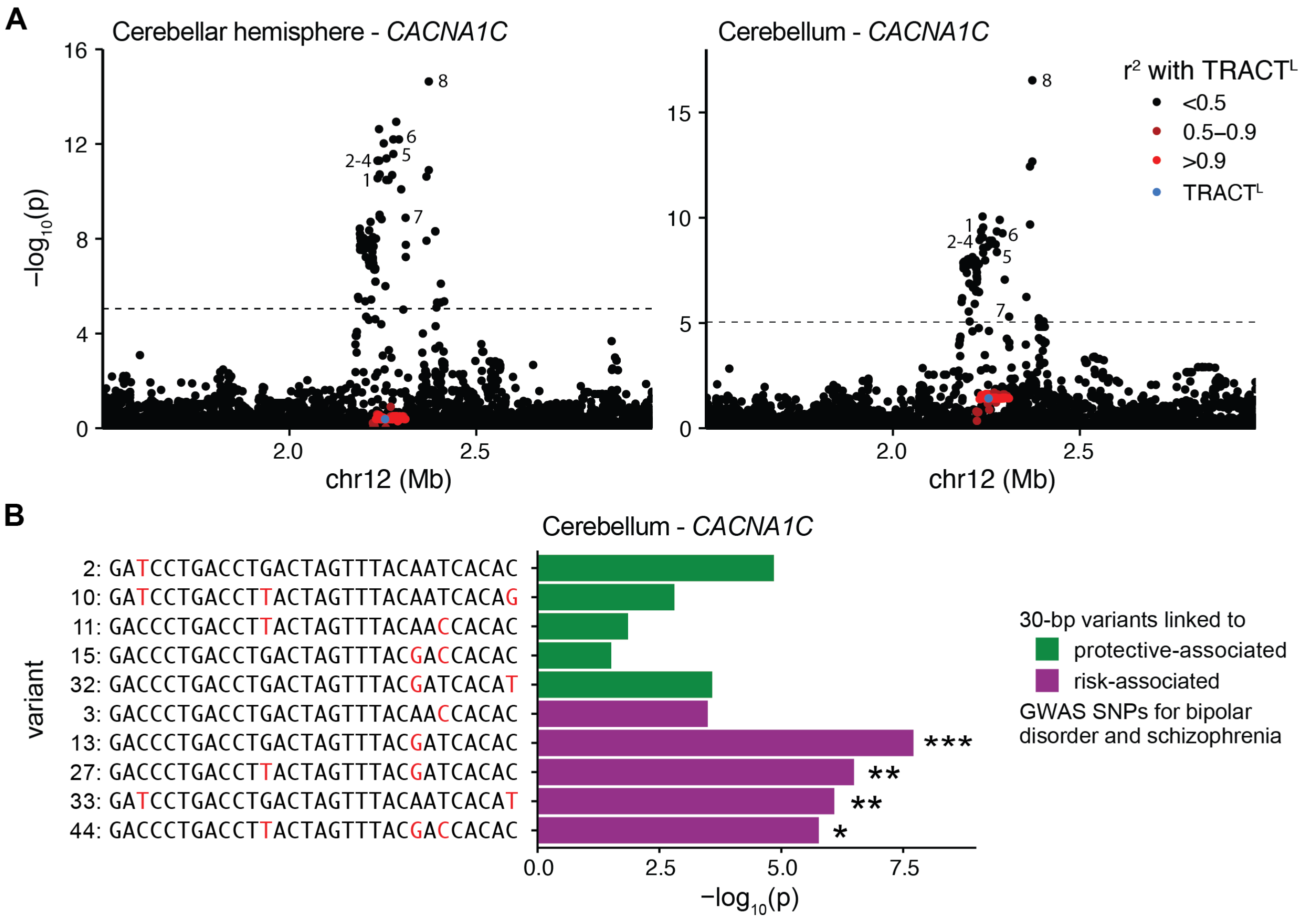

Figure S8: Neuropsychiatric GWAS SNPs and specific 30-bp variants are eQTLs for *CACNA1C* in the cerebellum. (A) The cerebellum was sampled twice in GTEx, labeled as either the cerebellar hemisphere (left) or the cerebellum (right). SNPs surrounding TRACT, but not TRACT^L itself (blue point) or SNPs in linkage disequilibrium with TRACT^L (red points), are eQTLs for *CACNA1C* expression in the cerebellar hemisphere (left) and cerebellum (right). SNPs labelled 1-7 are previously identified GWAS SNPs (Ferreira et al., 2008; Ripke et al., 2011; Sklar et al., 2011; Smoller et al., 2013; Ripke et al., 2013, 2014; Ruderfer et al., 2014; Song et al., 2018; Ikeda et al., 2019; Stahl et al., 2019; Lam et al., 2019). SNP 8 is an eQTL for *CACNA1C* in the cerebellum that is not in linkage disequilibrium with the GWAS locus. Fine mapping by GTEx suggests that the SNP 8 eQTL locus is distinct from the GWAS eQTL locus (Materials and Methods). 1: rs2007044, 2: rs1006737, 3: rs2159100, 4: rs4765905, 5: rs10744560, 6: rs1024582, 7: rs4765913, 8: rs886898. The dotted line indicates the significance threshold after Bonferroni correction for association with *CACNA1C* expression. (B) The proportion of particular 30-bp variants (13, 27, 33, and 44) are eQTLs for *CACNA1C* expression in the cerebellum. *: adjusted $p < 0.05$; **: adjusted $p < 0.01$; ***: adjusted $p < 0.001$.

References

1000 Genomes Project Consortium, 2015. A global reference for human genetic variation. *Nature*, **526**(7571):68–74.

Browning, S. R. and Browning, B. L., 2007. Rapid and accurate haplotype phasing and missing-data inference for whole-genome association studies by use of localized haplotype clustering. *American Journal of Human Genetics*, **81**(5):1084–1097.

Ferreira, M. A., O'Donovan, M. C., Meng, Y. A., Jones, I. R., Ruderfer, D. M., Jones, L., Fan, J., Kirov, G., Perlis, R. H., Green, E. K., *et al.*, 2008. Collaborative genome-wide association analysis supports a role for ANK3 and CACNA1C in bipolar disorder. *Nature Genetics*, **40**(9):1056–1058.

Ikeda, M., Takahashi, A., Kamatani, Y., Momozawa, Y., Saito, T., Kondo, K., Shimasaki, A., Kawase, K., Sakusabe, T., Iwayama, Y., *et al.*, 2019. Genome-wide association study detected novel susceptibility genes for schizophrenia and shared trans-populations/diseases genetic effect. *Schizophrenia Bulletin*, **45**(4):824–834.

Kronenberg, Z., Nolan, C., Porubsky, D., Mokveld, T., Rowell, W. J., Lee, S., Dolzhenko, E., Chang, P.-C., Holt, J. M., Saunders, C. T., *et al.*, 2025. The Platinum Pedigree: A long-read benchmark for genetic variants. *Nature Methods*, **22**(8):1669–1676.

Lam, M., Chen, C. Y., Li, Z., Martin, A. R., Bryois, J., Ma, X., Gaspar, H., Ikeda, M., Benyamin, B., Brown, B. C., *et al.*, 2019. Comparative genetic architectures of schizophrenia in East Asian and European populations. *Nature Genetics*, **51**(12):1670–1678.

Liao, W.-W., Asri, M., Ebler, J., Doerr, D., Haukness, M., Hickey, G., Lu, S., Lucas, J. K., Monlong, J., Abel, H. J., *et al.*, 2023. A draft human pangenome reference. *Nature*, **617**(7960):312–324.

Logsdon, G. A., Ebert, P., Audano, P. A., Loftus, M., Porubsky, D., Ebler, J., Yilmaz, F., Hallast, P., Prodanov, T., Yoo, D., *et al.*, 2025. Complex genetic variation in nearly complete human genomes. *Nature*, **644**(8076):430–441.

Lonsdale, J., Thomas, J., Salvatore, M., Phillips, R., Lo, E., Shad, S., Hasz, R., Walters, G., Garcia, F., Young, N., *et al.*, 2013. The Genotype-Tissue Expression (GTEx) project. *Nature Genetics*, **45**(6):580–585.

Marnetto, D. and Huerta-Sánchez, E., 2017. Haplostrips: Revealing population structure through haplotype visualization. *Methods in Ecology and Evolution*, **8**(10):1389–1392.

Ripke, S., Neale, B. M., Corvin, A., Walters, J. T., Farh, K. H., Holmans, P. A., Lee, P., Bulik-Sullivan, B., Collier, D. A., Huang, H., *et al.*, 2014. Biological insights from 108 schizophrenia-associated genetic loci. *Nature*, **511**(7510):421–427.

Ripke, S., O'Dushlaine, C., Chambert, K., Moran, J. L., Kahler, A. K., Akterin, S., Bergen, S. E., Collins, A. L., Crowley, J. J., Fromer, M., *et al.*, 2013. Genome-wide association analysis identifies 13 new risk loci for schizophrenia. *Nature Genetics*, **45**(10):1150–1159.

Ripke, S., Sanders, A. R., Kendler, K. S., Levinson, D. F., Sklar, P., Holmans, P. A., Lin, D. Y., Duan, J., Ophoff, R. A., Andreassen, O. A., *et al.*, 2011. Genome-wide association study identifies five new schizophrenia loci. *Nature Genetics*, **43**(10):969–976.

Ruderfer, D. M., Fanous, A. H., Ripke, S., McQuillin, A., Amdur, R. L., Gejman, P. V., O'Donovan, M. C., Andreassen, O. A., Djurovic, S., Hultman, C. M., *et al.*, 2014. Polygenic dissection of diagnosis and clinical dimensions of bipolar disorder and schizophrenia. *Molecular Psychiatry*, **19**(9):1017–1024.

Sklar, P., Ripke, S., Scott, L. J., Andreassen, O. A., Cichon, S., Craddock, N., Edenberg, H. J., Nurnberger, J. I., Rietschel, M., Blackwood, D., *et al.*, 2011. Large-scale genome-wide association analysis of bipolar disorder identifies a new susceptibility locus near ODZ4. *Nature Genetics*, **43**(10):977–983.

Smoller, J. W., Ripke, S., Lee, P. H., Neale, B., Nurnberger, J. I., Santangelo, S., Sullivan, P. F., Perlis, R. H., Purcell, S. M., Fanous, A., *et al.*, 2013. Identification of risk loci with shared effects on five major psychiatric disorders: A genome-wide analysis. *Lancet*, **381**(9875):1371–1379.

Song, J. H. T., Lowe, C. B., and Kingsley, D. M., 2018. Characterization of a human-specific tandem repeat associated with bipolar disorder and schizophrenia. *American Journal of Human Genetics*, **103**(3):421–430.

Stahl, E. A., Breen, G., Forstner, A. J., McQuillin, A., Ripke, S., Trubetskoy, V., Mattheisen, M., Wang, Y., Coleman, J. R. I., Gaspar, H. A., *et al.*, 2019. Genome-wide association study identifies 30 loci associated with bipolar disorder. *Nature Genetics*, **51**(5):793–803.
